# Effect of Heartfulness Meditation Among Long-Term, Short-Term and Non-meditators on Prefrontal Cortex Activity of Brain Using Machine Learning Classification: A Cross-Sectional Study

**DOI:** 10.7759/cureus.34977

**Published:** 2023-02-14

**Authors:** Anurag Shrivastava, Bikesh K Singh, Dwivedi Krishna, Prasanna Krishna, Deepeshwar Singh

**Affiliations:** 1 Biomedical Engineering, National Institute of Technology, Raipur, Raipur, IND; 2 Yoga Life Sciences, Swami Vivekananda Yoga Anusandhana Samsthana (S-VYASA), Bengluru, IND; 3 Welfare Harvesters, Banglore, IND; 4 Yoga and Life Sciences, Swami Vivekananda Yoga Anusandhana Samsthana (S-VYASA), Bangalore, IND

**Keywords:** classifiers, functional connectivity, machine learning, electroencephalograph (eeg), heartfulness meditation (hm)

## Abstract

Background

Meditation is a mental practice with health benefits and may increase activity in the prefrontal cortex of the brain. Heartfulness meditation (HM) is a modified form of rajyoga meditation supported by a unique feature called “yogic transmission.” This feasibility study aimed to explore the effect of HM on electroencephalogram (EEG) connectivity parameters of long-term meditators (LTM), short-term meditators (STM), and non-meditators (NM) with an application of machine learning models and determining classifier methods that can effectively discriminate between the groups.

Materials and methods

EEG data were collected from 34 participants. The functional connectivity parameters, correlation coefficient, clustering coefficient, shortest path, and phase locking value were utilized as a feature vector for classification. To evaluate the various states of HM practice, the categorization was done between (LTM, NM) and (STM, NM) using a multitude of machine learning classifiers.

Results

The classifier's performances were evaluated based on accuracy using 10-fold cross-validation. The results showed that the accuracy of machine learning models ranges from 84% to 100% while classifying LTM and NM, and accuracy from 80% to 93% while classifying STM and NM. It was found that decision trees, support vector machines, k-nearest neighbors, and ensemble classifiers performed better than linear discriminant analysis and logistic regression.

Conclusion

This is the first study to our knowledge employing machine learning for the classification among HM meditators and NM The results indicated that machine learning classifiers with EEG functional connectivity as a feature vector could be a viable marker for accessing meditation ability.

## Introduction

Meditation practice can be used for exercising the brain and therefore became a field of interest among researchers [1]. There are various meditation practices known globally, but HM and its effects have not been evaluated by neuroimaging techniques. HM is the modified form of raja yoga meditation which involves focusing on the heart rather than concentrating on breathing. The HM has a unique feature of yogic transmission which facilitates even a new practitioner to feel the effect of meditation in a very short duration. In HM, a practitioner is allowed to perform meditation along with the trainer (guru), who initiates the transmission as per procedure [2]. Research shows the benefits of HM in moderating vital heart parameters Heart rate, respiration rate, Systolic blood pressure [3], and stress level [4-6]. Studies carried out on HM during the COVID-19 pandemic situation indicate that HM helps to regulate overall anger, mood, depression [4], stress, and sleep quality [5]. In HM practice, participants are allowed to sit comfortably with their eyes closed and asked to contemplate the source of light within the heart. If the mind of the participants gets distracted, then they are advised to gently redirect their focus to the heart again. This meditation is more straightforward as participants do not have to focus on the breath or chant the mantras, which is a mandatory tool in several other forms of meditation [7]. There is very limited research examining the effect of HM on brain signals. Various forms of meditation studies explored the changes in different lobes of the brain using electroencephalogram (EEG). EEG is a non-invasive but powerful technique used for the analysis of the brain’s activity. It is captured from the scalp's surface with the help of electrodes which measure electrical signals generated by various actions of the brain. Traditionally, EEG signals are categorized into four frequency bands: delta (0-4 Hz), theta (4-8 Hz), alpha (8-12 Hz), and beta (12-30 Hz). Each band is a reflection of different activity patterns of the brain. EEG has a high temporal resolution, is relatively low cost, and is portable, therefore popular among researchers [8,9]. Functional connectivity is currently one of the most pertinent areas of study for neurological responses using EEG signal analysis. Understanding how information is processed can be gained by examining the modifications in node interactions brought about by meditation. In functional connectivity analysis, several features are calculated and stored in the matrix, representing the connectivity between each pair of nodes [10]. Functional connectivity can be used for analyzing cognitive activity [11], disorders like schizophrenia [12], depression [13], chronic pain [14], yoga, and meditation [15-17]. Therefore, we have used functional connectivity parameters in the present study such as Pearson correlation (r) [18], phase locking value (PLV) [19], clustering coefficient (CC), and shortest path (SP) [20]. Previous meditation reported an increase in functional connectivity of the brain during meditation as compared to the resting state [21]. Numerous studies indicate that connectivity increases in the brain's prefrontal cortex in experienced meditators compared to the non-meditators [22,23]. This study, therefore, focused on the prefrontal cortex. Different classifiers, including decision tree (DT), support vector machine (SVM), k-nearest neighbor (KNN), and ensemble classifier (EC), are employed in the meditation study to distinguish meditative and non-meditative states. [24]. Therefore, the purpose of this study is to make an objective measurement of HM by computing functional connectivity characteristics as a feature and selecting the best classifier that could distinguish between LTM, STM, and NM.

## Materials and methods

The objective of the study has been explained in PICO (population-intervention-comparison-outcome) format as shown in Table 1.

**Table 1:** PICO elements of the study PICO: population-intervention-comparison-outcome

Population	Intervention	Comparison	Outcome
Meditators (long term and short term) and non-meditators	Heartfulness meditation	Between meditators (long term and short term) and non-meditators	Machine learning classifiers accuracy

Participants

In total 45 (30 males and 15 female) participants in the age group between 20 and 45 years were recruited from Heartfulness Center, Bengaluru, India. Based on experience, participants were categorized into three groups: long-term meditator (LTM) having experience greater than five years, short-term meditator (STM) having experience less than three years, and non-meditator (NM) without meditation experience. The study was approved by the institutional ethics committee, Swami Vivekananda Yoga Anusandhana Samsthana (SVYASA), Bengaluru, India. Participants' demography characteristics are given in Table 2.

**Table 2:** Demographics of participants

S.no	Demographics	Gender	Long-term meditators	Short-term meditators	Non -meditators
1	Gender	Male	9	7	7
		Female	4	4	3
2	Age (years)	Male	32.54 ± 6.2	30 ± 7.5	28.43 ± 3.3
		Female	32.01 ± 6.4	29.45 ± 7.5	28.12 ±3.2
3	Meditation experience ( months)	Male+Female	98.71 ± 32.35	12.80 ± 6.48	----
4	Duration of practice/day (minutes)	Male+Female	76.07 ± 15.24	47.5 ± 20.36	----

Participants between the age of 20 and 45 years were included in the study. All the participants are mentally and physically healthy. People practicing other forms of meditation and diagnosed with mental and physical health issues were excluded from the study. Also, the participants selected are non-alcoholic, non-smokers, and not under any type of medication.

Experiment design

**Figure 1:**
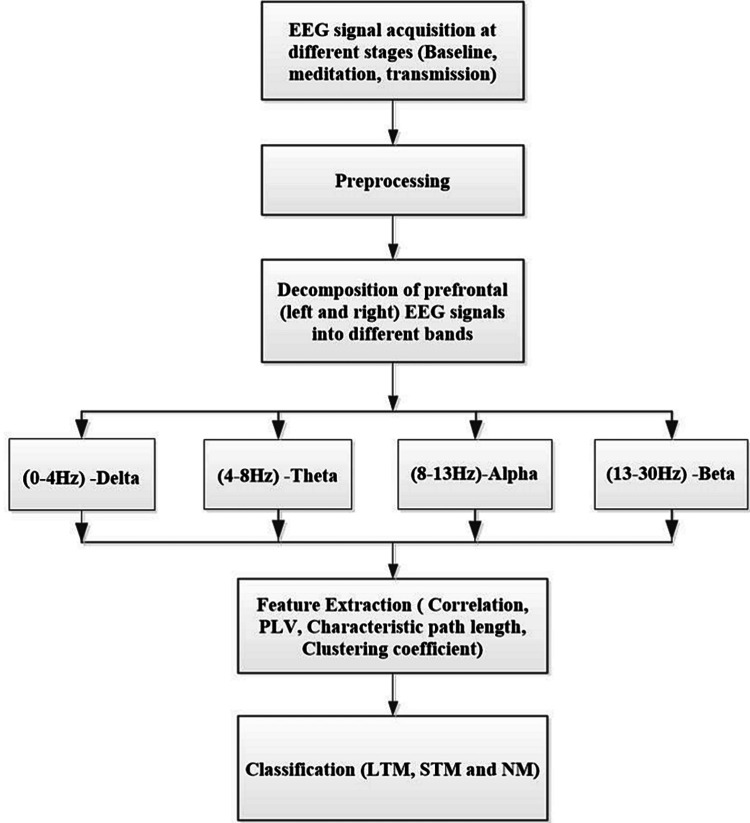
Flow diagram of EEG signal analysis EEG: electroencephalogram, LTM: long-term meditators, STM: short-term meditators, NM: non-meditaors, PLV: phase-locking value

The present study was an age-matched cross-sectional design. All participants' EEG data was collected and analyzed in the following stages as shown in Figure 1.

EEG Acquisition and Segmentation

The recording occurs at the cognitive neuroscience lab, SVYASA, Bengaluru, India. Each participant was assessed using 128 channels EEG system, (EGI geodesic transcranial electrical neuromodulation sensor GSN300), and data were recorded using EGI netstation (version 4.5.6) software. The sampling frequency was 250 Hz. The EEG recording took place in four states: Baseline state (5 minutes), where participants were instructed to relax with closed eyes. The second state was the meditation state (10 minutes), where participants were instructed to initiate HM practice, In the third state was transmission (10 minutes) participants were allowed to continue HM practice, and at the same time, an expert meditator (guru) starts the transmission to aid the practitioner, and the last state was post state (5 minutes) where participants were instructed to end meditation practice and relax.

Preprocessing and Band Extraction

**Figure 2:**
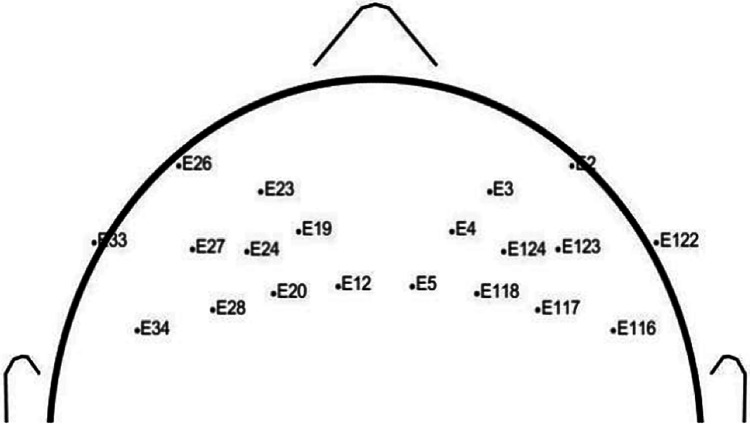
Electrode placements in prefrontal lobe of the brain

Preprocessing of the EEG signal was carried out in the EEGLAB toolbox (version 2021) [25]. The direct current noise was removed by applying a clean line. Further noises (muscular, ocular, and head movement) were removed by visual inspection and by applying Independent Component Analysis. The next stage after preprocessing was the segmentation of prefrontal cortex EEG signals into the left and right hemispheres. The electrodes corresponding to the prefrontal lobe were selected for this study (Figure 2). After segmentation, EEG bands (delta (0.3 - 4 Hz), theta (4-8 Hz), alpha (8-12 Hz), and beta (12-30 Hz)) were extracted using a bandpass filter. Functional connectivity was calculated for 20 electrodes. Prefrontal left and right 10 electrodes each.

Feature Selection

Functional connectivity features between each inter-region (left and right) electrode pair were calculated for classification. The features selected are, correlation coefficient (r), phase locking value (PLV), shortest path (SP), and clustering coefficient (CC).

Correlation coefficient (r): It is one of the basic features to measure the functional connectivity of the brain by accessing the degree of similarity between the pair of electrodes. It is the ratio of covariance between two signals and their respective variances. The correlation is calculated from equation below.

 $Cor(i,j)=(Cov(i,j))/\sqrt{(Var(i)Var(j))}$

Where Cov(i,j) is the cross-spectral density between two signals, and Var(i)and Var(j) are the auto spectral densities for signals i and j, respectively. The correlation value lies between +1 to -1, where +1 indicates that signals are perfect positive correlation, -1 indicates a perfect negative correlation, and 0 indicates that the two signals are perfectly uncorrelated [18,26] .

Phase-locking value (PLV): The PLV measures the phase synchronization between pair of electrodes in a functional brain network. The Hilbert transform will obtain the phase of the corresponding signals, and after that phase difference between the two signals will be calculated. The rage lies from +1 to 0, where + 1 represents perfect phase synchronization. [10] . For the electrode pair (i,j), the phase difference can be calculated from equation below.

$PLV_(i,j)=1/L \sum e^{(j\Theta (i,j)) }$ 

Where $\Theta (i,j))$ represents phase difference between electrodes i and j [19,27-28].

Characteristic path length (PL)/shortest path (SP): It is the measure of global efficiency calculated by the average of the shortest path between the nodes. Global efficiency is inversely proportional to the average shortest path. It represents the number of intermediate edges between pairs of electrodes that are responsible for information flow. There is more than one path possible between electrode pairs. Only the shortest path is taken into account because it is the fastest way for information transfer. It is used to measure functional connectivity [27] . The shorter path length represents better functional connectivity. The shortest path calculation was based on Dijkstra's algorithm. [29].

Clustering coefficient (CC): It measures the local segregation in a complex brain network by accessing the possibility of nodes from the cluster. Assuming there are three nodes, j, k, and l.suppose node j and k is connected to l, then the clustering coefficient reflects the probability of connection between j and k to form a triangle in a network. The clustering coefficient measures the speed of information processing and transmission within a network [26-27,29] .The clustering coefficient is calculated by equation below.

$C_j=1/(K_(j ) (K_j-1))\sum (w_{jk}w_{kl}w_{lm})^{\frac{1}{3}}$

Where k_i _is the degree of node and w_jk _,w_kl, _w_lm_ are the weights between nodes j and k, k and l, l and m, respectively [30].

Classification

The classification of selected features was performed by the classifier learner application in MATLAB R2018a. Further k-fold cross-validation method was used for system evaluation by segmenting the data set into training and testing data sets.

k-fold cross validation: It splits the data set into k groups. After that, it selects one group as the testing group and the other k-1 group as the training group. In this technique, each group will get a chance to become a testing group. In this study, 10-fold cross-validation is used for system evaluation. This means 10 times training and testing of data set is involved.

Classifiers and features: In this study, we have considered classification based on the meditation states (baseline, meditation, transmission) and classification based on the EEG signal band (delta, theta, alpha, beta, and gamma). The classification was performed for LTM vs NM and STM vs NM groups. Functional connectivity parameters are used as features given in Table 3.

**Table 3:** List of classifiers and features LTM: long-term meditators, STM: short-term meditators, NM: non-meditators

Classification group: state wise	Classifiers	Features
LTM vs NM	Decision tree, linear discriminate analysis, logistic regression, support vector machine, k nearest neighbor and ensemble	Correlation coefficient -(delta, theta, alpha,beta), phase locking value-(delta, theta, alpha,beta), shortest path- (delta, theta, alpha,beta) and clustering coefficient- (delta, theta, alpha,beta)
STM vs NM		
Classification group: band wise	Classifiers	Features
LTM vs NM	Decision tree, linear discriminate analysis, logistic regression, support vector machine, k nearest neighbor and ensemble	Correlation coefficient -(baseline, meditation, transmission, post), phase locking value-(baseline, meditation, transmission, post), shortest path- (baseline, meditation, transmission, post) and clustering coefficient- (baseline, meditation, transmission, post)
STM vs NM		

## Results

**Figure 3:**
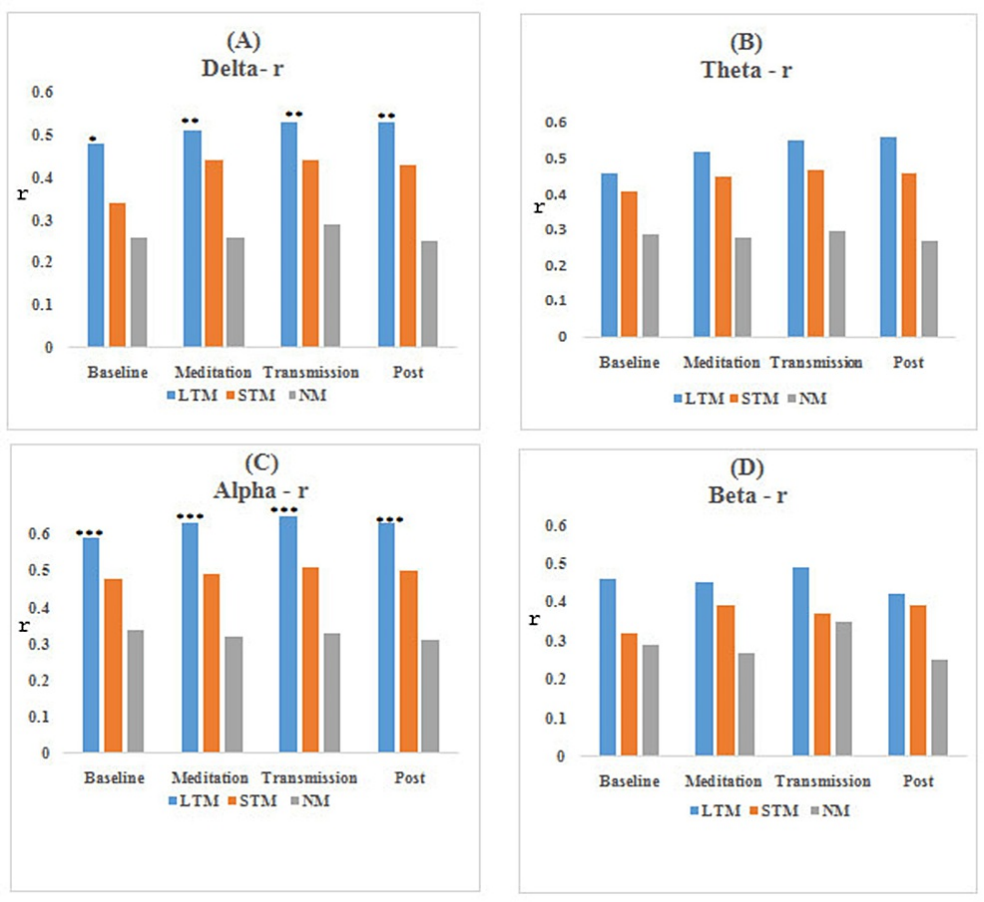
Average r values of EEG bands for different meditation states *compares NM group, *P<0.05, **P<0.01, and ***P<0.001. r: correlation coefficient, EEG: electroencephalogram, LTM: long-term meditators, STM: short-term meditators, NM: non-meditators

**Figure 4:**
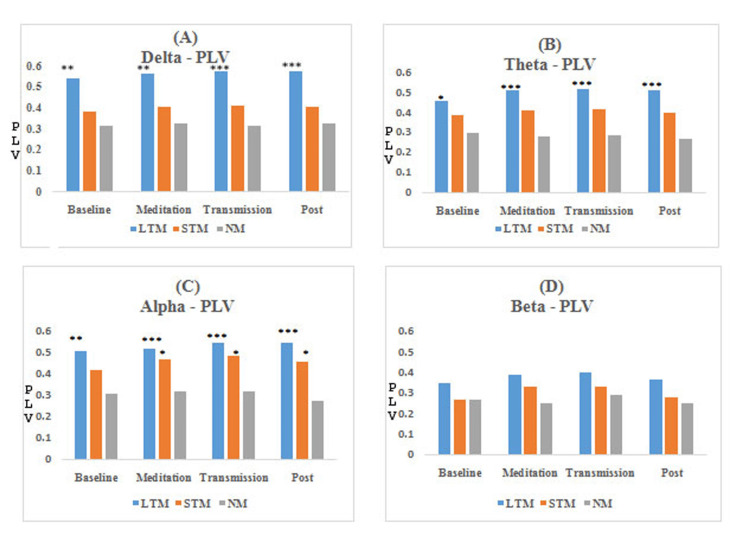
Average PLV values of EEG bands for different meditation states *compares NM group, *P<0.05, **P<0.01, and ***P<0.001. PLV: phase-locking value, EEG: electroencephalogram, LTM: long-term meditators, STM: short-term meditators, NM: non-meditators

**Figure 5:**
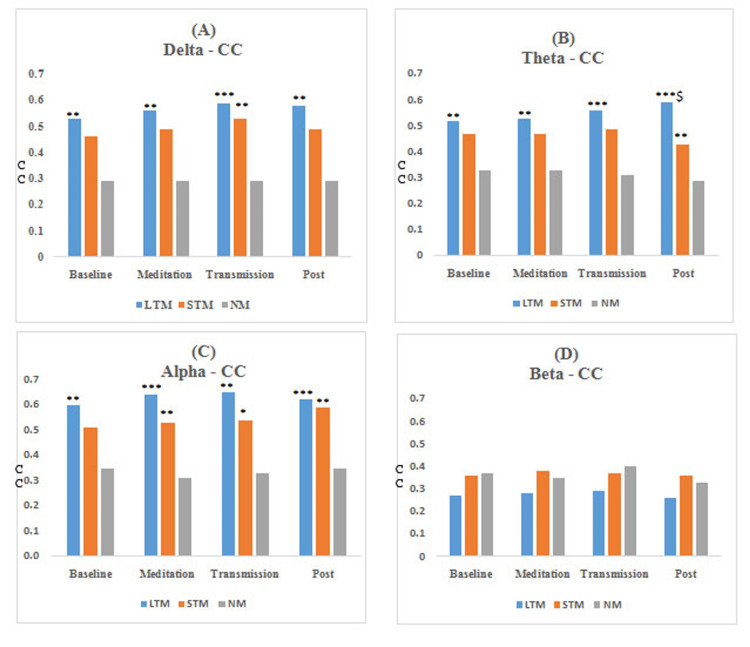
Average CC values of EEG bands for different meditation states *compares NM group, *P<0.05, **P<0.01, and ***P<0.001. CC: clustering coefficient, EEG: electroencephalogram, LTM: long-erm meditators, STM: short-term meditators, NM: non-meditators

**Figure 6:**
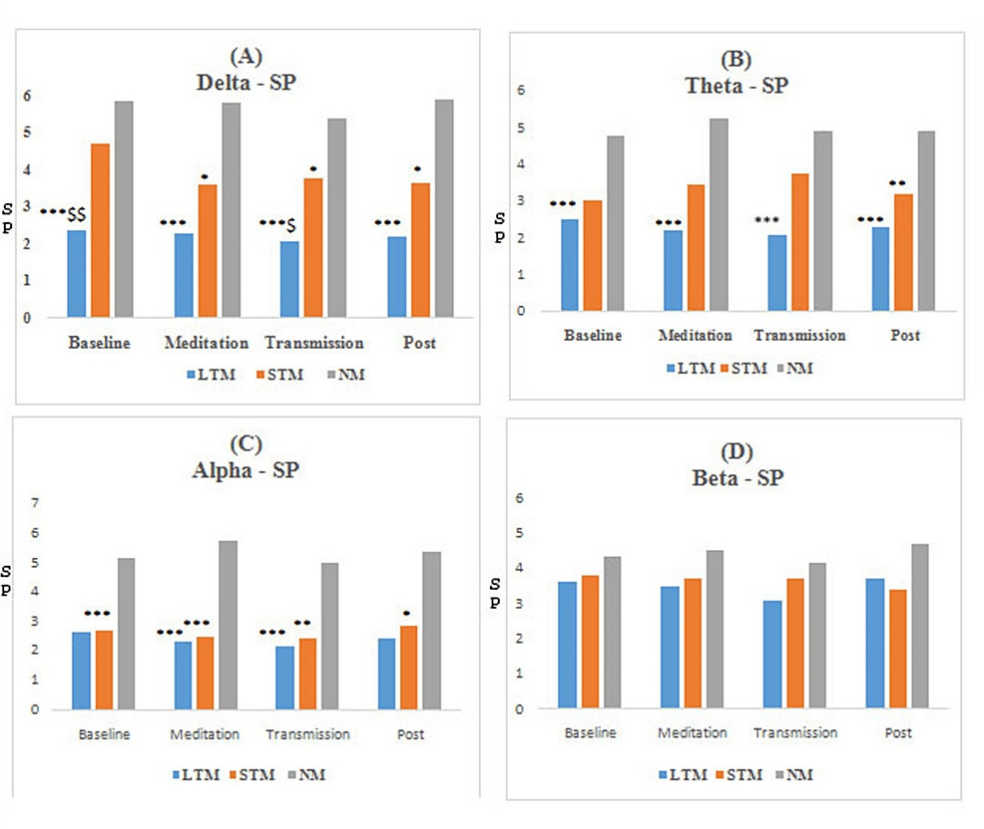
Average SP values of EEG bands for different meditation states *compares NM group and ^$^compares STM. *^$^P<0.05, **^$$^P<0.01, and ***^$$$^P<0.001. SP: shortest path, EEG: electroencephalogram, LTM: long-term meditators, STM: short-term meditators, NM: non-meditators

The results obtained from functional connectivity analysis of the prefrontal cortex along with the significance level for between-group comparison are shown in Figures 3-6. The graph indicates an increase in the value of r, PLV, CC, and decrement in SP in the meditation and transmission states, respectively, as compared to the baseline, which is an indicator of enhanced functional connectivity during meditation and transmission. In this study, these functional connectivity features were used to classify meditative and non-meditative states.

**Figure 7:**
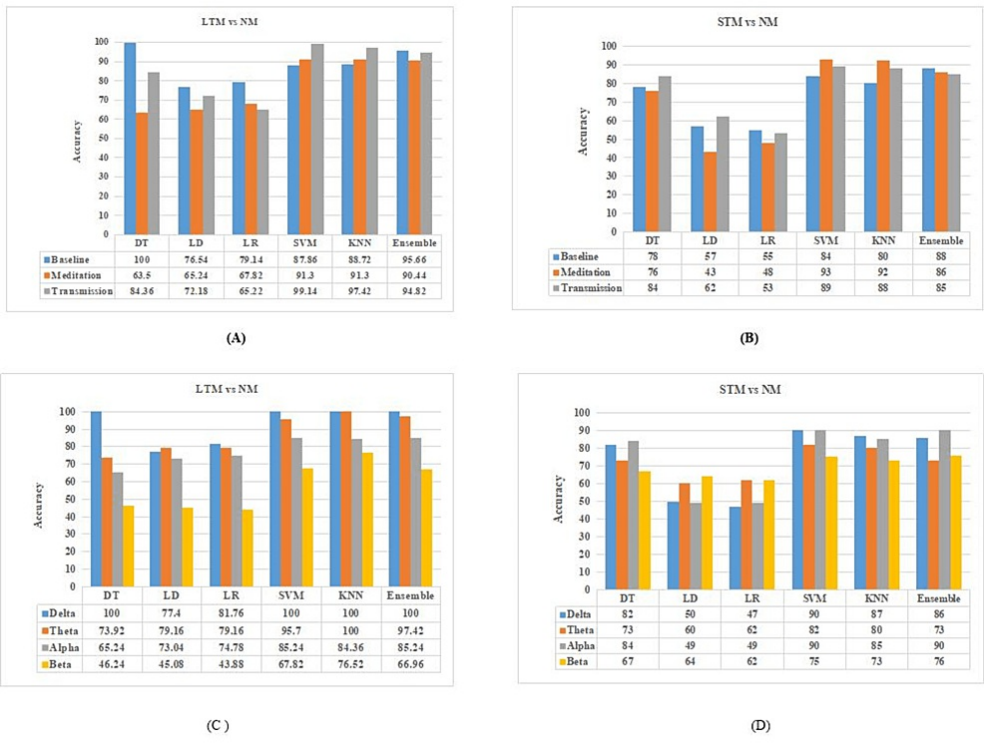
Group performance of classifiers (state wise and band wise) LD: linear discriminant, LR: logistic regression, DT: decision tree, SVM: support vector machine, KNN: k-nearest neighbor, LTM: long-term meditators, STM: short-term meditators, NM: non-meditators

The participants were categorized into three groups (LTM-13, STM-11, and NM-10), and six classifiers (Decision tree, Linear discriminate analysis, Logistic regression, Support vector machine, K nearest neighbor, and Ensemble) were compared based on their accuracy. The result of the percentage accuracy of various classifiers for state-wise classification is shown in Figures 7A, 7B. While performing state-wise classification between NM and LTM groups, the baseline achieved the highest accuracy for decision tree and ensemble classifiers of 100% and 95%, respectively. The meditation achieved the highest accuracy of 91% both for SVM and KNN. SVM and KNN classifiers during transmission achieved the highest accuracy of 99% and 97%, respectively. In contrast, the accuracy of LD and LR was lower during all the states.

The results of state-wise classification for the NM vs STM group reveal that for the baseline state, the highest accuracy was achieved for the ensemble classifier with an accuracy of 88%. The meditation state achieved the highest accuracy for SVM and KNN classifiers with an accuracy of 93% and 92%, respectively, and for the transmission state highest accuracy of 89% and 88% was achieved by employing SVM and KNN classifiers.

Now considering the band-wise classifier performance for STM vs NM group, the accuracy achieved by classifiers in the delta band was 100% for the decision tree, SVM, KNN, and ensemble classifiers, for theta band KNN, Ensemble, and SVM classifier shows the accuracy of 100%, 97%, and 95%, respectively. The SVM, ensemble, and KNN classifiers are in the alpha band. The performance of classifiers in the beta band was not satisfactory, with the highest accuracy of 76% achieved by KNN classifiers.

The result of band-wise classifier performance for the LTM vs NM group reveals that the highest accuracy achieved by the classifier in the delta band was 90% and 87% for SVM and KNN, respectively. The highest accuracy of 82% and 80% in the theta band was achieved by the SVM and KNN classifiers, respectively. In the alpha band, the SVM and ensemble classifiers achieved an accuracy of 90% each and the KNN classifier showed an accuracy of 85%. In this group also the performance of classifiers is not so satisfactory, with the highest accuracy of 76% achieved by the ensemble classifier. Figures 7C, 7D show the band-wise performance of classifiers.

## Discussion

This is the first study on HM employing machine learning to classify meditators and non-meditators. This study effectively categorizes participants into different classes, i.e., LTM, STM, and NM, using connectivity features. The connectivity feature results indicate an increase in value of the r, PLV, CC, and decrement in SP in the meditation and transmission states, respectively as compared to the baseline state, which is an indicator of enhanced functional connectivity during meditation and transmission. This result is aligned with the findings of [31-34], where focused attention (FA) meditation and integrative body-mind training and relaxation training respectively, enhance the connectivity features (r, PLV, and CC) while reducing SP thereby increasing global efficiency. The classifier results indicate that functional connectivity features were better for identifying the changes due to meditation. The comparison results among classifiers show that LTM vs NM was classified with higher accuracy as compared to STM vs NM. The results also indicated that SVM, KNN, DT, and Ensemble classifiers perform with better accuracy in most cases as compared to LDA and LR. DT performs better with the accuracy of 100% only in delta and baseline states while classifying LTM vs NM. The accuracy range of LTM vs NM ranges from 84% to 100%, whereas STM vs NM ranges from 80% to 93%.

The first limitation of this study is the small sample size for all three groups, which limits the generalization. The second limitation is the discomfort to the meditator during the EEG setup. Some other factors that can affect the results, such as heterogeneity in practice, and clinical history, were not taken into account in this study. Further study could use other neuro-imaging techniques with increased sample sizes for all the groups (LTM, STM, and NM) to support the findings of our study. Furthermore, a cardiovascular study employing an electrocardiogram (ECG) analysis technique can be incorporated to signify the coordination between the heart and brain while performing HM.

## Conclusions

This is the first study to our knowledge employing machine learning in HM. The shreds of evidence demonstrated a methodological pipeline for classification among meditators (LTM, STM) and NM to assess the impact of HM. The classifiers are compared based on their computational accuracy. The findings demonstrated the viability of DT, SVM, KNN, and Ensemble classifier with EEG functional connectivity as feature vectors for accessing meditation ability and their capability to accurately distinguish between meditative and non-meditative states. These classifiers can quantify meditation experience and meditation state effectively. The unique feature of HM 'Transmission' revealed distinct alterations in the prefrontal cortex of meditators. Furthermore, this study can be extended with different features and other classification techniques.
